# Inhibition of Integrin α_v_β_3_-FAK-MAPK signaling constrains the invasion of T-ALL cells

**DOI:** 10.1080/19336918.2023.2191913

**Published:** 2023-03-21

**Authors:** Lan Huang, Yao Zhu, Qinglin Kong, Xianmin Guan, Xiaoying Lei, Luying Zhang, Hui Yang, Xinyuan Yao, Shaoyan Liang, Xizhou An, Jie Yu

**Affiliations:** a Department of hematology and oncology, Children’s Hospital of Chongqing Medical University, National Clinical Research Center for Child Health and Disorders, Ministry of Education Key Laboratory of Child Development and Disorders, Chongqing Key Laboratory of Pediatrics, Chongqing, China; b Department of Hematology and Oncology, Chengdu Women’s and Children’s Central Hospital, School of Medicine, University of Electronic Science and Technology of China, Chengdu, China

**Keywords:** Cell invasion, central nervous system infiltration, FAK, integrin β3, T-ALL

## Abstract

The role of adhesion receptor integrin αvβ3 in T-ALL was unclear. Firstly, we performed quantitative real-time PCR to assess medullary expression of integrin β3(ITGB3) in T-ALL patients and high ITGB3 expression was relevant with the central nervous system leukemia(CNSL) incidence. Decreasing of cell invasion was observed in Jurkat and Molt4 treated with integrin αvβ3 specific antibody and inhibitor as well as cells with ITGB3 interference. Further, phosphorylation of FAK, cRAF, MEK and ERK decreased in cells with integrin αvβ3 inhibition or interference. Invasion decreased in T-ALL cells treated with FAK and ERK inhibitors. In conclusion, inhibition of integrin αvβ3 signals significantly limits the cell invasion of T-ALL cells.

## Introduction

T-cell acute lymphoblastic leukemia (T-ALL) is a highly aggressive hematological cancer caused by malignant immature T cells, representing 10–15% of pediatric and 25% of adult ALL cases [1]. It is clinically regarded as a high-risk type of leukemia and has a relapse rate of approximately 30% in children [2,3]. Although risk-adjusted chemotherapy has improved the outcomes of patients with ALL who present immature T-cell immunophenotype (i.e., T-ALL), patients suffering from induction failure, early relapse, and isolated central nervous system (CNS) relapse are still facing poor prognosis [4]. Extramedullary involvement (EMI) is a common manifestation seen in patients. During T-ALL diagnosis, infiltration of leukemic cells into the liver, mediastinum, CNS, and testis occur in approximately 30–50%, 8%, 2.5–5%, and 0.6% of patients, respectively [4–6]. EMI such as CNS leukemia (CNSL) and testicular leukemia (TL) is a factor that indicates a higher risk for patients with T-ALL [7]. Even though the high dose intravenous methotrexate (HDMTX) were applied in newly developed protocol, CNS or CNS/bone marrow (BM) relapse still contributed nearly 40% of relapse in T-ALL patients [8].

Integrin β3 (gene symbol: ITGB3) is a member of integrin family, which represents a group of immunoglobulin superfamily molecules that conduct cell adhesion, migration and related biology processes between cells and extracellular matrix (ECM) [9]. Several studies have elucidated that integrin β3 play important roles in tumors as a signal transducer that coordinate the signals from ECM into the cell to induce antiapoptotic effect, drug resistance and metastasis in tumor cells [10–13]. Although T-ALL is a malignant disease with intensive progression, the mechanism of T-ALL infiltration and the role of integrin β3, which was commonly expressed in lymphoblastic cells [14,15], in the infiltration progression are still unknown.

Hence, in this study, we used quantitative real-time PCR to determine the expression levels of integrin β3 and examine the relevance between integrin β3 expression and the infiltration manifestations of leukemia cells in T-ALL patients. Then, we explored the function of integrin β3 (ITGB3) in human T-ALL cell lines, Jurkat and MOLT-4 by determining the affect of ITGB3 inhibitors or ITGB3 RNA interference on the cell malignancy behavior and phosphorylation of downstream signal transducer of integrin, FAK-MAPK axis. Based on our findings, integrin β3 expression in the bone marrow T-ALL patients was relevant with the occurrence of CNS infiltration and inhibition of integrin β3 could decrease the adhesive and invasive ability of T-ALL cells.

## Materials and methods

### Patients and clinical samples

Primary bone marrow mononuclear cells (BMMNC) were obtained from 53 children diagnosed T-ALL who were hospitalized at the Children’s Hospital of Chongqing Medical University (CHCMU) from January 2016 to July 2019. Samples of all patients were obtained during diagnosis. T-ALL was diagnosed according to the WHO morphological, immunophenotypic, cytogenetic, and molecular (MICM) criteria for classifying hematopoietic and lymphoid tissue tumors 2008 and 2016, respectively [16,17]. All children with T-ALL received standardized chemotherapy and risk stratification evaluation following the Children’s Cancer and Leukemia Group (CCLG) ALL 2008 chemotherapy protocol and Chinese Children Cancer Group (CCCG) 2015 ALL chemotherapy protocol, respectively [18,19]. Data regarding their clinical manifestations and treatment responses were obtained from the standard electronic medical records system of the CHCMU. Extramedullary infiltration at diagnosis was defined following the definition provided by the CCLG2008 and CCCG2015 protocol, respectively and the diagnosis was made by professional pediatrician on the field of hematology in CHCMU. This study was approved by the Ethics Commission of the CHCMU（No.2015–23）. Informed consent form was obtained from all patients or from their guardians in case of minors.

Bone marrow samples (1–2 mL) were collected in tubes with EDTA anticoagulant and diluted with an equal volume of 0.01 M PBS. Mononuclear cells were separated on 5 mL of lymphocyte separation medium (Haoyang) by centrifugation at 1,000 rpm for 20 min at room temperature. The white cell layer was collected, washed twice with PBS, and resuspended in culture medium.

### Antibodies and reagents

Anti-FAK (D2R2E; Cell Signaling Technology), anti-pFAK (D20B1; Cell Signaling Technology), anti-integrin β3 (ab119992; Abcam), anti-MMP2 (ab92536; Abcam), anti-MMP9 (ab76003; Abcam), anti-integrin αv (ab179475; Abcam), anti-integrin α2b (ab134131; Abcam) and anti-GAPDH (ab181602; Abcam) antibodies were used as primary antibodies and a horseradish peroxidase (HRP)-conjugated goat anti-rabbit IgG (ZB2301; Zhongshan Biotechnology) was used as the secondary antibody for Western blotting. The anti-integrin αvβ3 antibody (ab78289; Abcam) and the anti-integrin α2bβ3 antibody (ab11027; Abcam) were used to inhibit the function of integrin αvβ3 and integrin α2bβ3 f*in vitro*, respectively. The anti-integrin β3 (ab119992; Abcam), anti-integrin αv (ab179475; Abcam), and anti-integrin α2b (ab134131; Abcam) were used for immunoprecipitation.

The specific integrin inhibitor cyclo(Arg-Gly-Asp-d-Tyr-Lys) peptides (cyclo(RGDyK)(S7844; Selleck Chemicals) was used for disturbing the function of integrin. The FAK specific inhibitor PF-573228 (S2013; Selleck Chemicals) and ERK inhibitor Magnolin (S9102; Selleck Chemicals) were used to study FAK-MAPK signaling.

### Cell lines and cell culture

The T-ALL cell lines used in this study, Jurkat(ATCC®TIB-152^TM^) and MOLT-4(ATCC®CRL-1582^TM^) were both obtained from an authorized distributor of ATCC in China (Zhongyuan Inc.). Both cell lines were cultured in RPMI 1640 medium (GIBCO) supplemented with 10% heat-inactivated fetal bovine serum (Cat no.10100147, GIBCO), 100 U/mL penicillin, and 100 μg/mL streptomycin (P1400, Solarbio). Cells were incubated at 37°C in a humidified atmosphere containing 5% CO_2_ and were passaged every 2–3 days following the instructions of ATCC. All cells were tested free of mycoplasma contamination.

### Lentivirus-mediated RNA interference

ITGB3 RNA interference (RNAi) lentivirus expressing the sequence CCACGTCTACCTTCACCAATA, which was designed from the ITGB3 cDNA sequence NM_000212.2, was purchased from Hanheng Biotech Co., Ltd. Lentivirus-mediated scramble siRNA was used as negative control. Virus solution with multiplicity of infection (MOI) 20 was added to 60–70% confluent Jurkat or MOLT-4 cells cultured in serum-free RPMI 1640 medium in T75 cell culture plates (IWAKI, Iwaki). After 6 h, the culture medium was replaced by complete medium and 800 ng/mL puromycin was supplemented into the medium the next day. The RNAi and RNAi control cells were harvested after 3 days, and ITGB3 expression was detected by real-time PCR and Western blot.

### Quantitative real-time RT-PCR

Total RNA was extracted and purified using TRIzol (Invitrogen) according to the manufacturer’s instructions. The RNA concentration was identified by NanoDrop^TM^ 2000 UV-Vis Spectrophotometer (ThermoFisher, Waltham, MA, USA) following the manufacturer’s instructions. Complementary DNAs (cDNAs) were synthesized from l μg of total RNA by quantitative reverse transcription PCR (qRT-PCR) using the TaKaRa RNA PCR Kit Ver. 2.1 (TaKaRa Bio). Real-time qRT-PCR was performed with the SYBR Green real-time qPCR Kit (TaKaRa Bio) using a StepOnePlus PCR instrument (Applied Biosystem, Waltham, MA, USA). The PCR products were subjected to a melting curve analysis and the mRNA expression levels were calculated using the 2^−ΔΔCt^ method [20]. GAPDH expression levels were used as internal controls and to normalize the expression levels. The primers used for real-time qRT-PCR are shown in Supplementary Table S1 in page 1. All assays were repeated three times in parallel.

### CCK-8 cell proliferation assays

The growth curve of Jurkat and Molt-4 cells was obtained using the Cell Counting Kit-8 (CCK-8) (Dojindo, Gaithersburg, MD, USA). Cells treated with cyclo(RGDyk) (0, 0.2, 0.4, 0.6, 0.8 and 1.0 mmol/L) and anti-αvβ3 antibodies (20 µg/mL) were seeded in a 96-well microplate at a density of 5,000 cells per well and incubated for 3 consecutive days. Colorimetric assays were performed according to the manufacturer’s instructions, and absorbance was measured at 450 nm using a microplate reader (Bio-Rad Laboratories, Hercules, CA, USA). Cells from each group were assayed three times in parallel. The control T-ALL cells treated with DMSO solution only were applied as 0 mmol/L cyclo(RGDyk) group, control group of antibodies treatment and the control group of kinase inhibitors treatment (all these with same treatment).

### Detection of apoptosis by flow cytometry

Apoptotic cells were stained with Annexin V-7AAD/APC apoptosis detection kit I (KeyGen Biotech, Beijing, CHINA) according to the manufacturer’s instructions. Stained cells were analyzed using a flow cytometer (BD Biosciences, San Jose, CA, USA) to detect the fluorescence levels. Early apoptotic cells were defined as cells with high Annexin V and low 7AAD signal (bottom right quadrant in the flow cytometry graph) and were measured as a percentage of the total cell count (1×10^6^ cells/mL). Cells from each group were assayed three times in parallel.

### Transwell invasion assays

Transwell assays were performed using Matrigel-coated Transwell chambers (Qiagen, Duesseldorf, Germany) with an 8.0 μm pore size. A total of 1 × 10^6^ cells were suspended in 200 uL of serum-free medium and seeded in the upper compartment of one chamber. The lower compartment was loaded with 800uL of complete culture medium containing 20% FBS. After incubation for 24 h at 37°C, the cells in the lower chambers were fixed with formaldehyde and counterstained with DAPI at room temperature for 30 min in the dark. The stained cells were then counted under a TE2000-U inverted fluorescence microscope (Nikon Inc., Chiyoda-ku, Tokyo, Japan). Each assay was repeated three times in parallel.

### Western blot

Total proteins were extracted using the Western and IP cell lysis kit (Beyotime, Songjiang, Shanghai, China) according to the manufacturer’s instructions and the protein concentrations were determined using the BCA protein assay kit (Beyotime, Songjiang, Shanghai, China). Total proteins (20 mg per lane) were separated on 10% SDS-PAGE gels and transferred onto PVDF membranes (Cat. No. 88518, Thermo Fisher, Waltham, MA, USA). The membranes were then blocked in 5% skim milk for 2 h at room temperature before being incubated with primary antibodies against ITGB3, FAK, pFAK, MMP2, MMP9, Bcl-2, Bax, and GAPDH (ITGB3, FAK, pFAK, MMP2, MMP9, Bcl-2 and Bax primary antibodies were diluted at 1:1000, GAPDH primary antibody was diluted at 1:2000) for overnight at 4°C. The membranes were then rinsed three times with TBS-T (Tris buffered saline with 0.1% Tween-20) and incubated with a secondary antibody solution for 2 h at room temperature. The secondary antibody was diluted at 1:10000 in skim milk. Protein bands were visualized using a new super ECL kit (Bio-Rad Laboratories, Hercules, CA, USA) according to the manufacturer’s instructions. The amount of protein in each band was quantified by software Quantity One 4.6.2 (Bio-Rad Laboratories, Inc., Hercules, CA, USA). All assays were repeated three times in parallel.

### Immunoprecipitation

The cells were harvested and total protein was extracted from T-ALL cells using cell lysis kit (ThermoFisher Waltham, WA, USA). A BCA protein assay kit (Beyotime, Shanghai, China) was used to determine the protein concentrations. Then, the acquired proteins (500–1000 ng) were incubated with specific antibodies or normal IgG at 4°C overnight. A total of 25 μL of A/G magnetic beads were added and incubated with the immune complexes for 1 h. Finally, the protein was eluted from the beads for western blotting. This assay was repeated three times in parallel.

### Statistical analyses

Statistical analyses were performed using the IBM SPSS statistics software version 19.0 (IBM Corporation, Armonk, NY, USA). Experimental and clinical data were expressed as means ± standard deviation (SD). Statistical significance was analyzed using Student’s *t*-test and one-way analysis of variance (ANOVA) for the data following Gaussian distribution, and Mann-Whitney U-test or Kruskal–Wallis H test for the data not following the Gaussian distribution. *P* < .05 was considered to be statistically significant.

## Results

### High ITGB3 expression was associated with CNS infiltration of T-ALL patients

Previous studies have reported a close relationship between integrin β3 and tumor progression in various malignancies [21–24], so we collected the BMMNC which were obtained from 53 children with T-ALL, the characteristics of patients are shown in Supplementary Table 2 in page 2 and 3. And we retrospectively analyzed the relevance between ITGB3 expression level and the manifestations of EMI (Table 1) and other clinical manifestations (Supplementary Table 3 in page 4) at the time of diagnosis in patients with T-ALL. The results revealed that the ITGB3 expression was not associated (*P* > .05) with bone, kidney, mediastinum, skin, testis infiltration and others, but higher ITGB3 expression showed correlation with the occurrence of CNS infiltration, which was diagnosed as central nervous system leukemia (CNSL). Based on these results, elevated levels of integrin β3 during diagnosis may be considered as a marker for CNS infiltration.

**Table 1.** ITGB3 expression in patients grouped by leukemic infiltration indexes.

Index		N	Median[P25, P75] of ITGB3 expression	P
**CNS Involvement**				
	No	33	0.002102[0.001127, 0.002968]	0.042*
	Yes	20	0.003162[0.001517, 0.024198]	
**Bone Infiltration**				
	No	20	0.001976[0.001257, 0.002811]	0.300
	Yes	33	0.002595[0.001016, 0.013381]	
**Mediastinal Infiltration**				
	No	31	0.002245[0.000990, 0.012778]	0.639
	Yes	22	0.002592[0.001502, 0.006134]	
**Renal Infiltration**				
	No	35	0.002291[0.001003, 0.006395]	0.940
	Yes	18	0.002445[0.001450, 0.009879]	
**Skin manifestations**				
	No	25	0.002175[0.001016, 0.006312]	0.831
	Yes	28	0.002492[0.001257, 0.007903]	

CNS, central nervous system.

### In vitro inhibition of integrin β3 decreased the invasion of T-ALL cells

**Figure 1.**
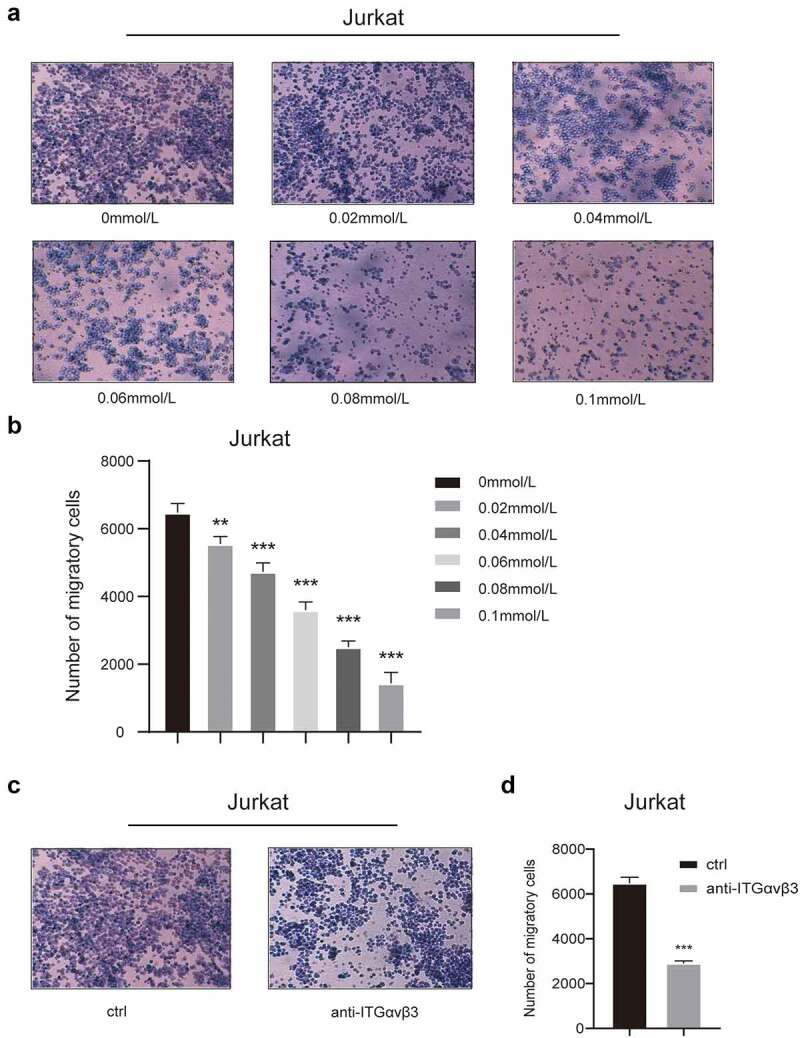
**The cell invasion of Jurkat cells with*in vitro* integrin β3 inhibition. (a)** The transwell analysis of invasion of Jurkat cells treated with cyclo(rgdyk) in concentration ladder of 0, 0.2, 0.4, 0.6, 0.8, 1.0 mmol/L after 24 hours. The migrated cells were stained with crystal violet. **(b)** Column graph of invasive Jurkat cells treated with cyclo(rgdyk) in concentration ladder of 0, 0.2, 0.4, 0.6, 0.8, 1.0 mmol/L after 24 hours detected by transwell analysis. ***P<.01, ***P<.001. **(c)** The transwell analysis of invasion of control Jurkat cells(ctrl) and those treated with integrin αvβ3 specific antibodies(anti-ITGαvβ3). The migrated cells were stained with crystal violet. **(d)** Column graph of invasion of control Jurkat cells(ctrl) and those treated with integrin αvβ3 specific antibodies(anti-ITGαvβ3) detected by transwell analysis. **P<.001.

To investigate the impact of integrin β3 on T-ALL cell, Jurkat and Molt-4 cell lines obtained from T-ALL patient was used to mimic T-ALL cells *in vitro*. Cyclo(RGDyk), a specific inhibitor for integrins, and specific integrin β3 were used to inhibit the function of integrin β3 *in vitro* to confirm the impact of integrin β3 on T-ALL cells. Then, cell growth curves (Supplementary Fig1A, 1B, Supplementary Fig2A, 2B) in 72 h were detected. The cyclo(RGDyk) could decrease the cell growth of T-ALL cells when concentration increased, however, the function block antibody did not change the growth curve of T-ALL cells significantly (*P* > .05). Further, cell apoptosis (Supplementary Fig1C, 1D, 1E, 1F, Supplementary Fig2C, 2D, 2E, 2F) was leveled through Annexin V staining by flow cytometry detection, and no significant changes of apoptosis rate were induced by either cyclo(RGDyk) or function block antibody to integrin β3. Transwell assays (Figure 1a-d, Supplementary Fig3A~D) were performed to detect the cell invasion. As the concentration of cyclo(RGDyk) increased, the cell invasiveness was decreased significantly in both cell lines (*P* < .05). Also, the addition of antibody to integrin β3 could decrease the cell invasion of both cell lines (*P* < .05). Thus, these results suggested that inhibition of integrin β3 *in vitro* decreased the cell invasiveness of T-ALL cells.

### ITGB3 knockdown decreased the invasion of the T-ALL cells

**Figure 2.**
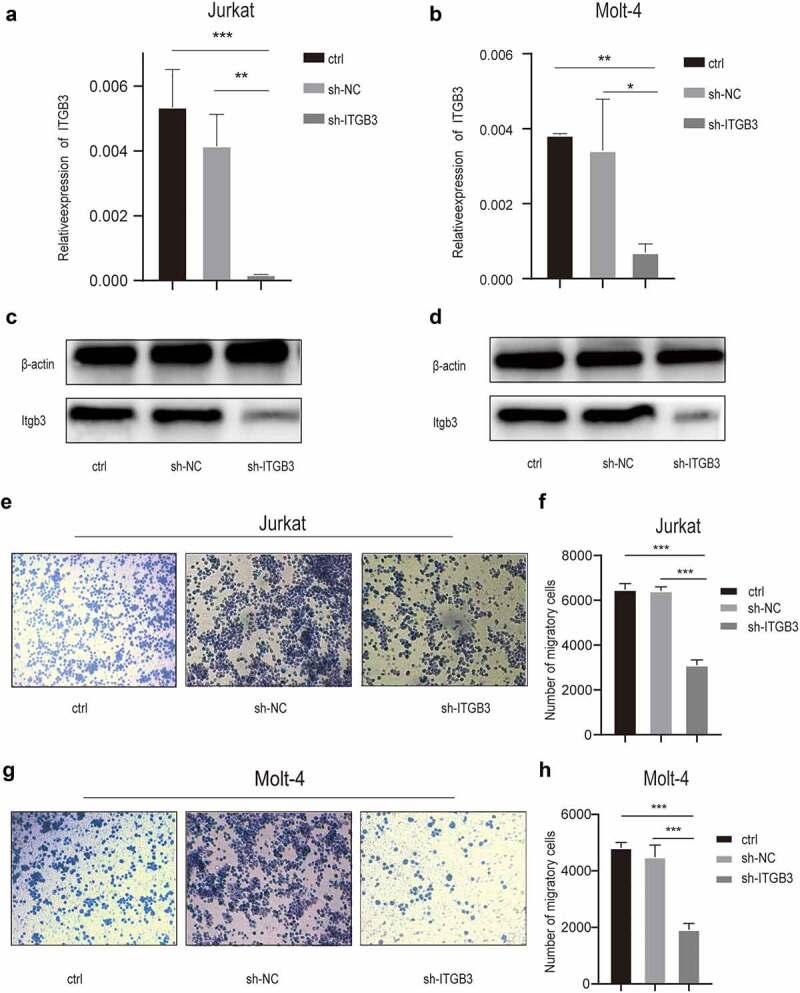
**Interference of ITGB3 expression decreased the invasiveness of Jurkat cells and Molt-4 cells.(a)** the expression of ITGB3 mRNA in common Jurkat cells (ctrl), cells infected by scramble control RNAi virus (sh-NC) and ITGB3 RNAi virus interfered cells (sh-ITGB3). The relative expression level was presented as the ratio of integrin β3 and β-actin, which was applied as endogenous control. ***P<.001, ***P<.01. (**b**) The expression of ITGB3 mRNA in common MOLT-4 cells (ctrl), blank control cells infected by scramble control RNAi virus (sh-NC) and ITGB3 RNAi virus interfered cells (sh-ITGB3). The expression was **P<.001, ***P<.05. **(c)** The expression of integrin β3 in Jurkat(ctrl), sh-NC and sh-ITGB3 cells detected by Western blot. **(d)** The expression of integrin β3 in MOLT-4 (ctrl), sh-NC and sh-ITGB3 cells detected by Western blot. The expression of β-actin was used as internal control. **(e)** The migratory cell counts of Jurkat (ctrl), sh-NC and sh-ITGB3 cells in transwell assays. **(f)** The column graph of migratory cell counts of Jurkat, BC and shITGB3 cells. ***P<.001. **(g)** The migratory cell counts of MOLT-4 (ctrl), sh-NC and sh-ITGB3 cells in transwell assays. **(h)** The column graph of migratory cell counts of MOLT-4 (ctrl), sh-NC and sh-ITGB3 cells. ***P<.001.

To further investigate the role of integrin β3 in Jurkat cells, we infected Jurkat and MOLT-4 cells with a lentivirus that express siRNAs targeting ITGB3. Following infection with the ITGB3-targeting lentivirus, ITGB3 expression was determined in Jurkat (Figure 2a,c) and MOLT-4 (Figure 2b,d) cells by qRT-PCR and Western blot, respectively. The lentivirus-mediated siRNA significantly reduced the ITGB3 expression (*P* < .05) in T-ALL cells.

We performed cell growth assays to evaluate the effect of ITGB3 knockdown in both T-ALL cell lines (Supplementary Fig4A, 4B). However, ITGB3 knockdown showed no significant effect on cell growth, which was similar to the cells treated by integrin β3 antibodies. Then, we assessed the apoptosis levels by flow cytometry (Supplementary Fig4C, 4E) and found that ITGB3 knockdown did not increase the cell apoptosis rate compared with cells infected with the blank control viruses (*P* > .05, Supplementary Fig4D, 4F). Furthermore, the invasiveness between different groups of T-ALL cells was assessed by Transwell assays (Figure 2e,g), and the results showed that the knockdown of ITGB3 significantly decreased the invasion of Jurkat cells (*P* < .001, Figure 2f-h). Overall, the effects of ITGB3 knockdown on the behavior of T-ALL cells suggested that integrin β3 might function as a possible cell invasiveness modulator. In contrast with the results of integrin inhibitor, the growth of T-ALL cells was not affected by interfering with the expression of integrin β3.

### Integrin αv partner with integrin β3 to regulate the invasion of T-ALL cells

**Figure 3.**
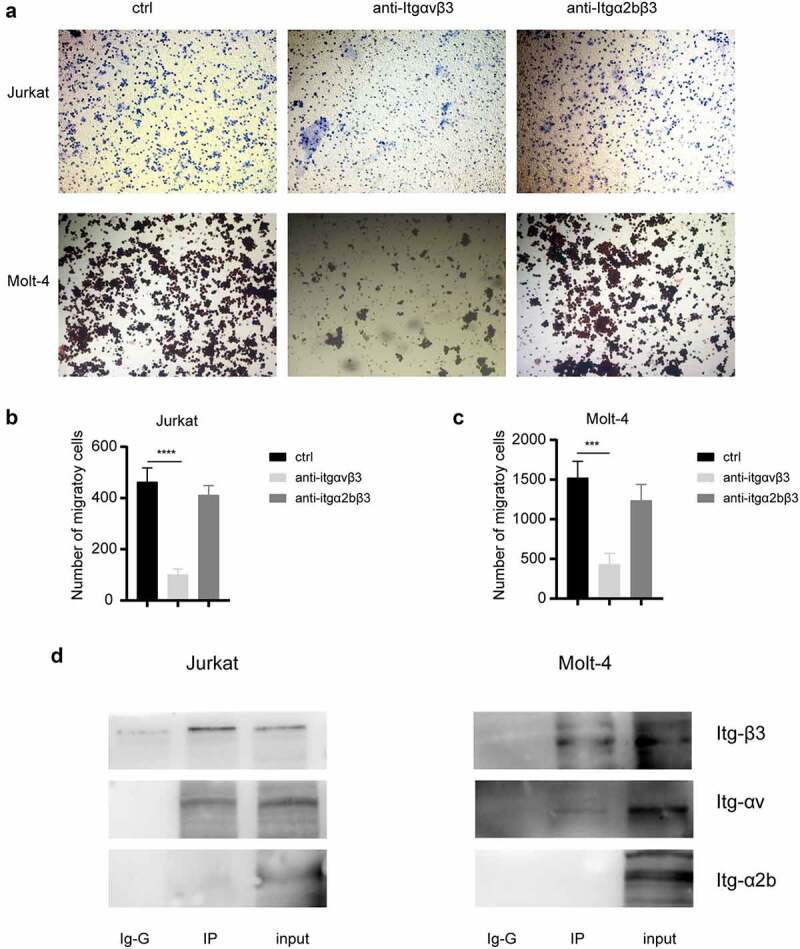
**Integrin αV not integrin α2b interact with integrin β3 to regulate the migration of T-ALL cells. (a)** The transwell assays for T-ALL cell lines, Jurkat and Molt-4, treated with solution control (ctrl), anti-integrin αvβ3 antibodies (anti-itgαvβ3) and anti-integrin α2bβ3 antibodies (anti- itgα2bβ3), respectively. **(b, c)** The statistical comparison of migrated T-ALL cell count (B: Jurkat, C: Molt-4) between three groups in transwell assays in (A). ***P<.001. **(d)** Western blot for the immunoprecipitation of α chain integrin interact with integrin β3. IgG and input represent the negative and positive control, respectively.

Two α subunit of integrin, integrin αv [21] and α2b [22] were reported to partner with integrin β3 in previous studies. In this study, we performed transwell assays to detect the invasiveness of Jurkat and MOLT4 cells treated with either integrin αvβ3 or integrin α2bβ3 specific antibodies (Figure 3a). The statistics of the invasive cell counts (Figure 3b,c) showed that the integrin αvβ3 specific antibodies could significantly decrease the invasiveness of both Jurkat and MOLT4 cells compared to those treated with solution only; however, the integrin α2bβ3 specific antibodies treatment did not modify the invasiveness of T-ALL cells significantly, which suggested integrin αv might be the partner of integrin β3 to regulate the invasiveness in T-ALL cells. To confirm the partner of integrin β3, we further performed the immunoprecipitation assay (Figure 3d), and the results showed that integrin β3 interact with integrin αv but not integrin α2b in T-ALL cells. Together, the integrin β3 partner with integrin αv to regulate the invasiveness of T-ALL cells.

### Integrin β3 activated FAK signaling to promote the invasion of T-ALL cells

To understand the molecular mechanism through which integrin β3 regulated cell invasion, we performed Western blot to detect the protein levels of the tumor cell invasiveness inducers, MMP2 and MMP9 (Supplementary Fig5A, 5C), in ITGB3 interfered cells and control cells. The results showed no significant differences in the expression of these genes in both cell lines.

**Figure 4.**
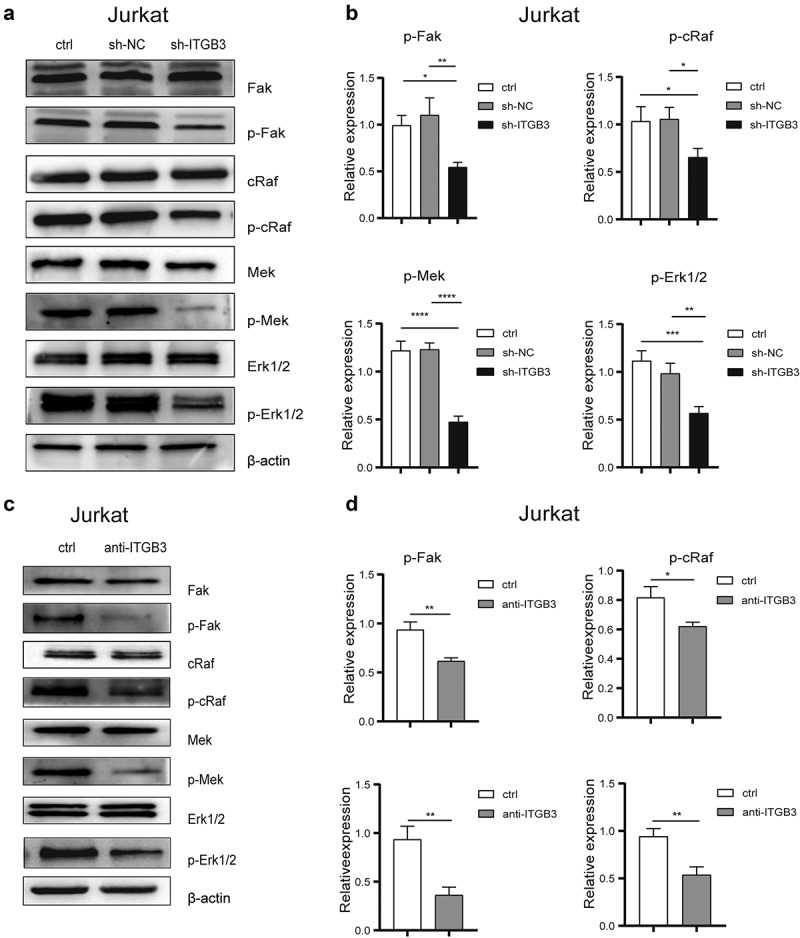
**Interference of ITGB3 expression decreased the phosphorylation of FAK-MAPK pathway in Jurkat cells. (a)** The total protein expression level and phosphorylation level of FAK, cRAF, MEK and ERK1/2 in Jurkat (ctrl), sh-NC and sh-ITGB3 cells detected by immunoblot. The expression of actin was applied as internal control. **(b)** The column graph of relative expression level of phosphorylation of FAK, cRAF, MEK and ERK1/2 in Jurkat (ctrl), sh-NC and sh-ITGB3 cells detected by immunoblot. The relative expression levels were calculated by the ratio of density of phosphorylation band and total protein band. **(c)** The total protein expression level and phosphorylation level of FAK, cRAF, MEK and ERK1/2 in control (ctrl) and Jurkat cells treated with ITGB3 antibodies (anti-ITGB3). The expression level was detected by immunoblot. The expression of actin was applied as internal control. **(d)** The column graph of relative expression level of phosphorylation of FAK, cRAF, MEK and ERK1/2 in control (ctrl) and Jurkat cells treated with integrinαvβ3 antibodies (anti-ITGB3) detected by immunoblot. The relative expression levels were calculated by the ratio of density of phosphorylation band and total protein band. ***P<.05, ***P<.01, **P<.001.

FAK was identified as a cytoplasmic protein tyrosine kinase that acts downstream of the integrin family members, including integrin β3, during tumor invasion [23–26]. We performed Western blot analysis to detect the levels of phosphorylated FAK and the downstream MAPK cascade in Jurkat and MOLT-4 cells to better understand the signal transduction pathway underlying integrin β3-mediated cell invasion of T-ALL cells. The results revealed a significant decrease in the levels of phosphorylated FAK, cRAF, MEK and ERK following ITGB3 knockdown (Figure 4a,b, Supplementary Fig6A, 6B), as well as in cells treated with cyclo(RGDyk) (Supplementary Fig5B, 5D) or function block ITGB3 antibodies (Figure 4c,d, Supplementary Fig6C, 6D).

**Figure 5.**
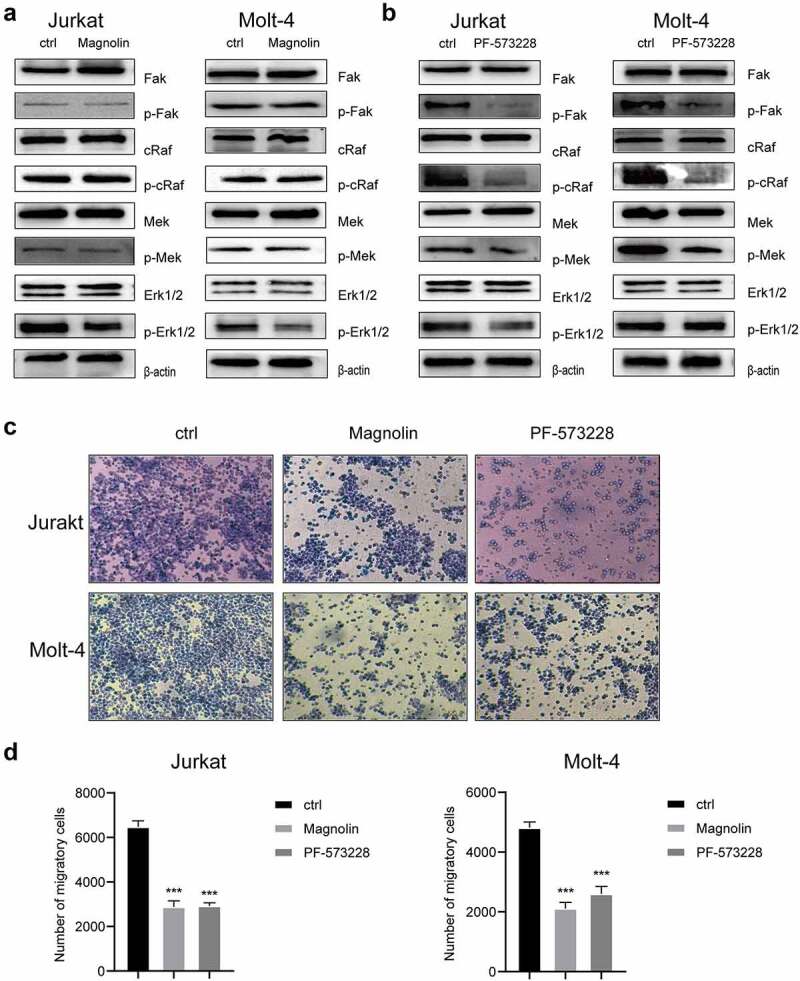
**Inhibition of FAK and ERK1/2 could inhibited T-ALL cell invasion. (a)** The total protein expression level and phosphorylation level of FAK, cRAF, MEK and ERK1/2 in control (ctrl) T-ALL cells and T-ALL cells treated with ERK1/2 specific inhibitor, Magnolin. The expression level was detected by immunoblot. The expression of actin was applied as internal control. **(b)** The total protein expression level and phosphorylation level of FAK, cRAF, MEK and ERK1/2 in control (ctrl) and T-ALL cells treated with FAK specific inhibitor, PF573228. The expression level was detected by immunoblot. The expression of actin was applied as internal control. **(c)** The transwell analysis of invasion of control (ctrl) T-ALL cells and T-ALL cells treated with Magnolin or PF-573228 after 24 hours. The migrated cells were stained with crystal violet. **(d)** The column graphs of migratory cell counts of control T-ALL cells (ctrl) and T-ALL cells treated with Magnolin or PF-573228. ***P<.001.

Moreover, we treated T-ALL cells with FAK inhibitor PF-573228 and ERK inhibitor Magnolin to confirm the involvement of signaling pathway downstream of integrin β3. The phosphorylation of FAK, cRAF, MEK were down-regulated by PF573228 (Figure 5a) whereas the phosphorylation of ERK was down-regulated only by direct ERK inhibitor Magnolin (Figure 5b). Then, Transwell assays (Figure 5c) were performed to assess the invasiveness in PF-573228 and Magnolin treated T-ALL cells. The invasion of both Jurkat and MOLT-4 cells were significantly decreased after treated with PF-573228 or Magnolin (Figure 5d), which was similar to the effect of ITGB3 knockdown. Based on these results, it was confirmed that FAK-MAPK pathway acts as a signal transduction pathway downstream integrin β3 to decrease the invasiveness of T-ALL cells.

## Discussion

Integrin β3, also known as CD61, is commonly considered as a marker of platelets or megakaryocytes and is involved in the terminal differentiation of platelets [27]. As shown in previous studies, integrin β3 plays a role in proliferation [28,29], invasion [10,11], metastasis [10,28,30] and chemoresistance [31] in various types of malignancies, including acute myeloid leukemia [32,33]. In this study, we investigated the expression of integrin β3 in bone marrow samples obtained from children with T-ALL. Relevance analysis of clinical manifestations and integrin β3 expression showed that higher expression of integrin β3 was correlated with the occurrence of CNS infiltration (Table 1). Some previous studies have proven the effectiveness to hamper the adhesion processes, the first events in the metastatic cascade and tumor spreading, of different cancer cells differentially expressing some integrins by using RGD cyclic peptide or integrin-specific blocking antibodies [34,35] and in line with literature studies our results showed that integrin-specific inhibitor cyclo(RGDyk) and function block antibody to integrin-β3 significantly decreased the cell invasion, as well as in the T-ALL cells in which the ITGB3 gene expression was knocked down by lentivirus-induced RNA interference. Furthermore, we detected the activation FAK-MAPK pathway, and applied the FAK inhibitor PF-573228 and the ERK inhibitor Magnolin to treat the T-ALL cells. Similarly to BCR-ABL driven chronic myelogenous leukemia (CML) cells in which the treatment with imatinib (BCR-ABL tyrosine kinase activity inhibitor) was able to abrogate the huge MAPK pathway activation [36–38], PF573228 and Magnolin down-regulated the activation of MAPK pathway in T-ALL cells and constrained the cell invasion, which suggested that FAK-MAPK acts as a signal pathway downstream integrin β3 to regulate invasion of T-ALL cells. Based on our results, integrin β3 activates FAK-MAPK to modulate the invasiveness of T-ALL cells, thereby potentially being able to influence CNS infiltration.

Although integrin β3 was identified as an inducer of invasion or metastasis in solid tumors [10,39,40], study by Yi *et al*. [32] showed that integrin αvβ3 antagonized tyrosine kinase inhibitor (TKI) activity of sorafenib in patients with Fms-related tyrosine kinase 3 (Flt3) mutated AML. Furthermore, in the study conducted by Miller *et al*. [41], integrin β3 signaling blocked the differentiation of progenitors that promote tumorigenesis in AML. However, in this study, integrin β3 activated FAK-MAPK signaling to promote the invasion of T-ALL cells, showed a role similar to that described in solid tumors but not like that in AML, which is another type of hematologic malignant. Further, T-ALL cells harbor a strong invasive potential that underlies many EMI manifestations in patients with T-ALL at diagnosis, and these conditions probably provided integrin β3 signaling an opportunity to regulate cell–ECM interactions and cell invasiveness in a manner similar to its function in solid tumors. In our study, although integrin β3 inhibition or interference hindered the invasion of T-ALL cells, we did not observe significant changes in MMP2 or MMP9 expression in cells with integrin β3 knockdown. This in turn suggests a different mechanism that may regulate the interaction between T-ALL cells and the ECM.

FAK is a tyrosine kinase that plays critical roles in downstream signaling of integrins [42] and is essential for cancer cell adhesion, invasion, and motility [43]. FAK synergistically functions with Philadelphia chromosome (Ph)-induced Abl signaling to promote the development of Ph+ ALL [44,45]. Therefore, FAK has been proposed as a therapeutic target for chemotherapy-resistant lymphoblastic leukemia [45]. Our results suggested integrin β3 might contribute a potential link between FAK signaling and CNSL in patients with T-ALL, supporting the hypothesis of FAK targeting therapeutic options, considering that 30%–40% of T-ALL relapses occur in the CNS [8].

Based on the correlation between ITGB3 expression and CNSL occurrence, we mainly discussed the regulation of cell invasion in T-ALL cells. However, cell growth disturbance was also induced in T-ALL cells by β-integrin inhibitor cyclo(RGDyk) treatment but not in cells with integrin β3 specific antibody treatment or ITGB3 interference. Considering that the integrin inhibitor cyclo(RGDyk) was a type of RGD peptide, which was a category of broad-spectrum integrin inhibitor, could inhibit a few types of integrins such as integrin αvβ5 [46,47], α5β1 [47] and other adhesion molecules such as vitronectin [48] besides integrin αvβ3 [49], these results might be explained by the inhibition of other integrins.

Organ infiltration including central nervous system (CNS) involvement was considered as risk factors for prognosis of T-ALL just years ago [50–52]. However, recent cohort study suggested that CNS state has no prognostic on pediatric T-ALL [53], even was not relevant with the CNS relapse [54]. In this study, we noticed that expression of ITGB3 in BMMNC at diagnosis was positively relevant with occurrence of CNS involvement and negatively relevant with risk stratification. However, the expression level of ITGB3 was not related with any other reported risk factors, such as peripheral WBC at diagnosis, age or gender. Considering there were just a few cases were recruited in this study and all cases were from only one center, we thought the relevance between ITGB3 expression and prognosis need further investigation based on a cohort with more cases from multicenter.

Collectively, our data indicated that down-regulation of integrin β3-FAK-MAPK axis could effectively decrease the invasion of T-ALL cells. Considering the correlation between integrin β3 and CNS infiltration, which was noticed in our retrospective analysis, strategies targeting integrin β3-FAK-MAPK axis might be an option for the development of alternative therapies to treat T-ALL patients with CNSL. In the further study, the gaps between the *in vitro* cell line results and the CNS infiltration *in vivo* needs to be investigated.

## Supplementary Material

Supplemental Material

## Data Availability

The authors confirm that the data supporting the findings of this study are available within the article and its supplementary materials. The detailed data that support the findings of this study are available on request from the corresponding author, [XA]. The data are not publicly available due to their containing information that could compromise the privacy of authors.
