# Supplementary material for: Inhibition of Integrin αvβ3-FAK-MAPK signaling constrains the invasion of T-ALL cells
Source: Cell Adh Migr. 2023 Mar 21;17(1):1–14. doi: 10.1080/19336918.2023.2191913 (PMC10038045; doi:10.1080/19336918.2023.2191913)
Supplement: Supplemental Material [file KCAM_A_2191913_SM9144.zip › 20221231_Revised Supplementary Materials_clean.docx]

**Supplementary Table 1. Primers used in real-time PCR analysis.**

| Gene  Symbol | Primer Sequences  (5'-3') | Template  Sequences |
| --- | --- | --- |
| ITGB3 | Forward: CCCTGCTCATCTGGAAACTC  Reverse: TTGGTGAAGGTAGACGTGGC | NM_000212.2 |
| GAPDH | Forward: GGTCACCAGGGCTGCTTTTA  Reverse: GAGGGATCTCGCTCCTGGA | NM_002046.5 |

**Supplementary Table 2.** **Characteristics of patients.**

| **Index** | | **N (%)** | **Median [P25, P75]** |
| --- | --- | --- | --- |
| **Gender** |  | **53(100)** |  |
|  | Male | 42(79.2) |  |
|  | Female | 11(20.8) |  |
| **Age** | | **53(100)** | **99[67, 121]** |
|  | <10 | 39(73.6) | 84[60.5, 102.5] |
|  | ≥10 | 14(26.4) | 146[132.75, 166] |
| **WBC** | | **53(100)** | **75.58[13.52, 359.87]** |
|  | <50 | 21(39.6) | 9.68[3.65, 21.73] |
|  | ≥50 | 32(60.4) | 338.38[82.38, 492.76] |
| **Risk Stratification** | | **53(100)** |  |
|  | Intermediate | 36(67.9) |  |
|  | High | 17(32.1) |  |
| **Karyotype** | | **51(100)^a^** |  |
|  | 46, XY or XX | 45(88.2) |  |
|  | Abnormal | 6(11.8) |  |
| **Hepatomegaly** | | **53(100)** |  |
|  | No | 4(7.5) |  |
|  | Yes | 49(92.5) |  |
| **Splenomegaly** | | **53(100)** |  |
|  | No | 5(9.4) |  |
|  | Yes | 48(90.6) |  |
| **Lymph adenomegaly** | | **53(100)** |  |
|  | No | 0(0) |  |
|  | Yes | 53(100) |  |
| **Bone infiltration** | | **53(100)** |  |
|  | No | 20(37.7) |  |
|  | Yes | 33(62.3) |  |
| **Mediastinal Infiltration** | | **53(100)** |  |
|  | No | 31(58.5) |  |
|  | Yes | 22(41.5) |  |
| **Renal Infiltration** | | **53(100)** |  |
|  | No | 35(66.0) |  |
|  | Yes | 18(34.0) |  |
| **CNS Involvement** |  | **53(100)** |  |
|  | No | 33(62.3) |  |
|  | Yes | 20(37.7) |  |
| **Testicular Leukemia** | | **53(100)** |  |
|  | No | 51(96.2) |  |
|  | Yes | 2(3.8) |  |
| **Skin symptoms** |  | **53(100)** |  |
|  | No | 25(47.2) |  |
|  | Yes | 28(52.8) |  |
| **ITGB3 expression** |  | **53(100)** | **0.002395[0.001164, 0.006479]** |

a, two patients died of leukemia complications onset before the evaluations were performed; WBC, count of leukocyte in peripheral blood at diagnosis; BM, bone marrow; MRD, minimal residual disease; CR, complete remission; CNS, central nervous system.

**Supplementary Table 3. ITGB3 expression in patients grouped by clinical indexes.**

| **Index** | | **N** | **Median[P25, P75] expression**  **level of ITGB3** | **P** |
| --- | --- | --- | --- | --- |
| **Gender** | |  |  |  |
|  | Male | 42 | 0.002445[0.001292, 0.006134] | 0.669 |
|  | Female | 11 | 0.002029[0.001003, 0.012778] |  |
| **Age (years)** | |  |  |  |
|  | <10 | 39 | 0.002595[0.001276, 0.006395] | 0.285 |
|  | ≥10 | 14 | 0.002129[0.000607, 0.009778] |  |
| **WBC (×10^9^/L)** | |  |  |  |
|  | <50 | 21 | 0.002679[0.001318, 0.031061] | 0.164 |
|  | ≥50 | 32 | 0.002175[0.001164, 0.003162] |  |
| **Risk stratification** | |  |  |  |
|  | Intermediate | 36 | 0.002595[0.001650, 0.023507] | 0.021* |
|  | High | 17 | 0.001307[0.000880, 0.002960] |  |
| **Karyotype** |  |  |  |  |
|  | 46, XY or XX | 45 | 0.002494[0.001307, 0.006479] | 0.597 |
|  | Abnormal | 6 | 0.001790[0.000489, 0.039387] |  |

**Supplementary Figure legends**

- **Supplementary Figure 1. The cell growth curve and apoptosis rate of Jurkat cells with *in vitro* integrin β3 inhibition. (A)** The 450nm absorbance of Jurkat cells treated with cyclo(RGDyk) in concentration ladder of 0, 0.2, 0.4, 0.6, 0.8, 1.0mmol/L in 3 days. **(B)** The 450nm absorbance of control Jurkat cells(ctrl) and those treated with integrin αvβ3 specific antibodies(anti-ITGB3). **(C)** The flow cytometric analysis of apoptotic ratio of Jurkat cells treated with cyclo(RGDyk) in concentration ladder of 0, 0.2, 0.4, 0.6, 0.8, 1.0mmol/L after 24 hours. The apoptotic cells were marked with positive Annexin-V staining (APC-A). **(D)** Column graph of apoptosis ratio of Jurkat cells treated with cyclo(RGDyk) in concentration ladder of 0, 0.2, 0.4, 0.6, 0.8, 1.0mmol/L after 24 hours detected by flow cytometric analysis. **(E)** The flow cytometric analysis of apoptotic ratio of control Jurkat cells(ctrl) and those treated with integrin αvβ3 specific antibodies(anti-ITGB3). The apoptotic cells were marked with positive Annexin-V staining. **(F)** Column graph of apoptosis ratio of control Jurkat cells(ctrl) and those treated with integrin αvβ3 specific antibodies(anti-ITGB3) detected by flow cytometric analysis.

**Supplementary Figure 2. The cell growth curve and apoptosis rate of MOLT-4 cells with *in vitro* integrin β3 inhibition.** **(A)** The 450nm absorbance of MOLT-4 cells treated with cyclo(RGDyk) in concentration ladder of 0, 0.2, 0.4, 0.6, 0.8, 1.0mmol/L in 3 days. *P<0.05. **(B)** The 450nm absorbance of control MOLT-4 cells(ctrl) and those treated with integrin αvβ3 specific antibodies(anti-ITGB3). **(C)** The flow cytometric analysis of apoptotic ratio of MOLT-4 cells treated with cyclo(RGDyk) in concentration ladder of 0, 0.2, 0.4, 0.6, 0.8, 1.0mmol/L after 24 hours. The apoptotic cells were marked with positive Annexin-V staining. **(D)** Column graph of apoptosis ratio of MOLT-4 cells treated with cyclo(RGDyk) in concentration ladder of 0, 0.2, 0.4, 0.6, 0.8, 1.0mmol/L after 24 hours detected by flow cytometric analysis. **(E)** The flow cytometric analysis of apoptotic ratio of control MOLT-4 cells(ctrl) and those treated with integrin αvβ3 specific antibodies (anti-ITGB3). The apoptotic cells were marked with positive Annexin-V staining. **(F)** Column graph of apoptosis ratio of control MOLT-4 cells (ctrl) and those treated with integrin αvβ3 specific antibodies (anti-ITGB3) detected by flow cytometric analysis.

**Supplementary Figure 3. The cell migration of MOLT-4 cells with *in vitro* integrin β3 inhibition.** (A) The transwell analysis of migration of MOLT-4 cells treated with cyclo(RGDyk) in concentration ladder of 0, 0.2, 0.4, 0.6, 0.8, 1.0mmol/L after 24 hours. The migrated cells were stained with crystal violet. (B) Column graph of migration of MOLT-4 cells treated with cyclo(RGDyk) in concentration ladder of 0, 0.2, 0.4, 0.6, 0.8, 1.0mmol/L after 24 hours detected by transwell analysis. **P<0.01, ***P<0.001. (C) The transwell analysis of migration of control MOLT-4 cells (ctrl) and those treated with integrin αvβ3 specific antibodies (anti-ITGB3). The migrated cells were stained with crystal violet. (D) Column graph of migration of control MOLT-4 cells (ctrl) and those treated with integrin αvβ3 specific antibodies (anti-ITGB3) detected by transwell analysis. ***P<0.001.

**Supplementary Figure 4.** **Effect of ITGB3 interference on the growth and apoptosis of Jurkat and MOLT-4 cells. (A)** The 450nm absorbance of the 3-day growth curve of Jurkat(ctrl), sh-NC and sh-ITGB3 cells. **(B)** The 450nm absorbance of the 3-day growth curve of MOLT-4 (ctrl), sh-NC and sh-ITGB3 cells. **(C)** The flow cytometric analysis of apoptotic ratio of Jurkat(ctrl), sh-NC and sh-ITGB3 cells. **(D)** The column graph of apoptotic ratio of Jurkat(ctrl), sh-NC and sh-ITGB3 cells. **(E)** The flow cytometric analysis of apoptotic ratio of MOLT-4 (ctrl), sh-NC and sh-ITGB3 cells. **(F)** The column graph of apoptotic ratio of MOLT-4 (ctrl), sh-NC and sh-ITGB3 cells.

**Supplementary Figure 5. The expression of MMP2 and MMP9, and the phosphorylation of FAK-MAPK cascade in T-ALL cells treated with RGDyk (A)** The expression of MMP2 and MMP9 in JURKAT (ctrl), sh-NC and sh-ITGB3 cells detected by immunoblot. The expression of β-actin was used as internal control. **(B)** The total protein expression level and phosphorylation level of FAK, cRAF, MEK and ERK1/2 in JURKAT cells treated with cyclo(RGDyk) in concentration ladder (0, 0.2, 0.4, 0.6, 0.8, 1.0mmol/L). The expression level was detected by immunoblot. The expression of β-actin was used as internal control. **(C)** The expression of MMP2 and MMP9 in MOLT-4 (ctrl), sh-NC and sh-ITGB3 cells detected by immunoblot. The expression of β-actin was used as internal control. **(D)** The total protein expression level and phosphorylation level of FAK, cRAF, MEK and ERK1/2 in MOLT-4 cells treated with cyclo(RGDyk) in concentration ladder (0, 0.2, 0.4, 0.6, 0.8, 1.0mmol/L). The expression level was detected by immunoblot. The expression of β-actin was used as internal control.

**Supplementary Figure 6. Interference of ITGB3 expression decreased the phosphorylation of FAK-MAPK pathway in MOLT-4 cells.** **(A)** The total protein expression level and phosphorylation level of FAK, cRAF, MEK and ERK1/2 in MOLT-4 (ctrl), sh-NC and sh-ITGB3 cells detected by immunoblot. The expression of actin was applied as internal control. **(B)** The column graph of relative expression level of phosphorylation of FAK, cRAF, MEK and ERK1/2 in MOLT-4 (ctrl), sh-NC and sh-ITGB3 cells detected by immunoblot. The relative expression levels were calculated by the ratio of density of phosphorylation band and total protein band. **(C)** The total protein expression level and phosphorylation level of FAK, cRAF, MEK and ERK1/2 in control (ctrl) and MOLT-4 cells treated with ITGB3 antibodies (anti-ITGB3). The expression level was detected by immunoblot. The expression of actin was applied as internal control. **(D)** The column graph of relative expression level of phosphorylation of FAK, cRAF, MEK and ERK1/2 in control (ctrl) and MOLT-4 cells treated with integrin αvβ3 antibodies (anti-ITGB3) detected by immunoblot. The relative expression levels were calculated by the ratio of density of phosphorylation band and total protein band. *P<0.05, **P<0.01, ***P<0.001.
