# Supplementary figures and images for: Inhibition of Integrin αvβ3-FAK-MAPK signaling constrains the invasion of T-ALL cells
Source: Cell Adh Migr. 2023 Mar 21;17(1):1–14. doi: 10.1080/19336918.2023.2191913 (PMC10038045; doi:10.1080/19336918.2023.2191913)

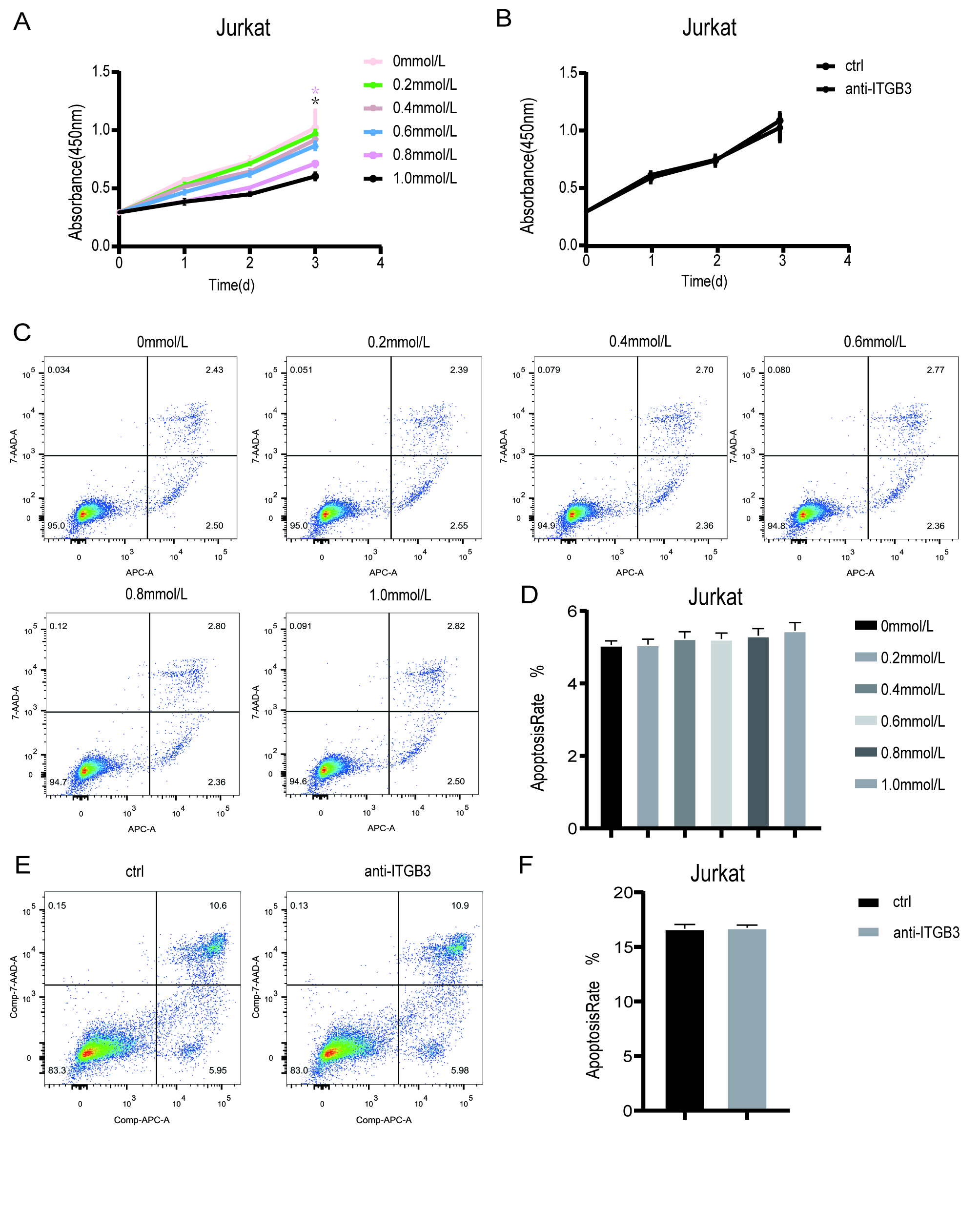

Supplement: Supplemental Material [file KCAM_A_2191913_SM9144.zip › Supplementary Figure1_for review_1.tif]

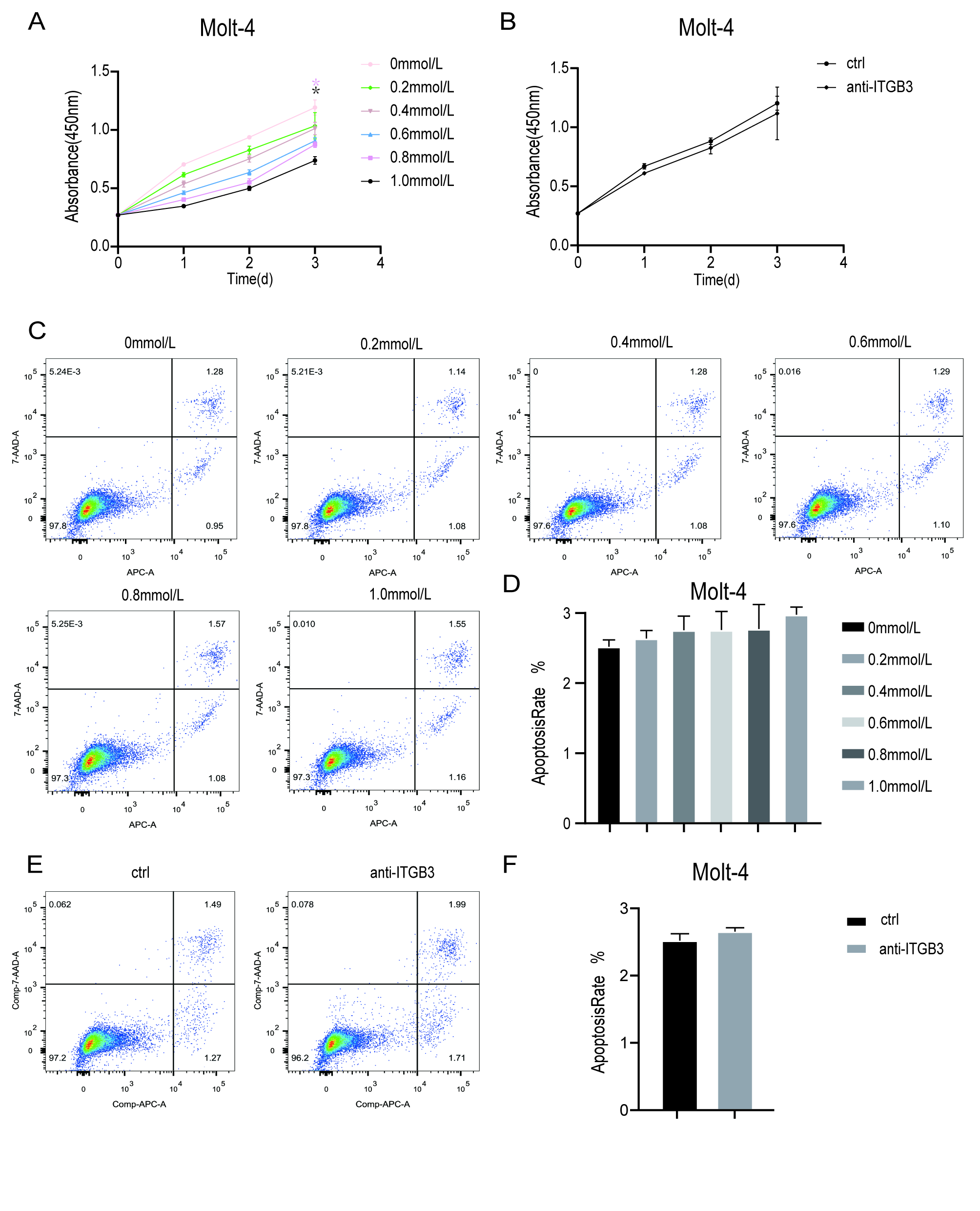

Supplement: Supplemental Material [file KCAM_A_2191913_SM9144.zip › Supplementary Figure2_for review.tif]

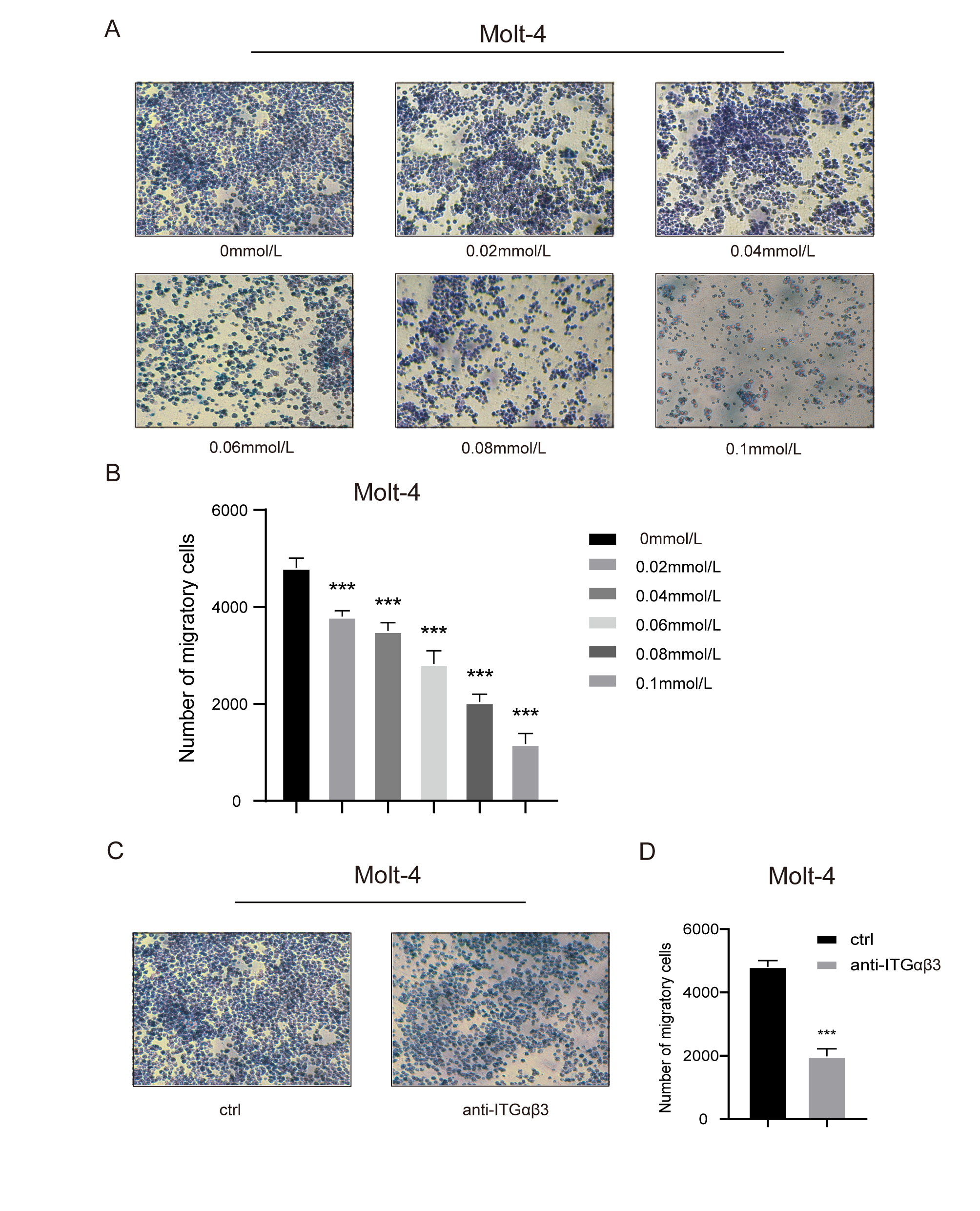

Supplement: Supplemental Material [file KCAM_A_2191913_SM9144.zip › Supplementary Figure3_for review_1.tif]

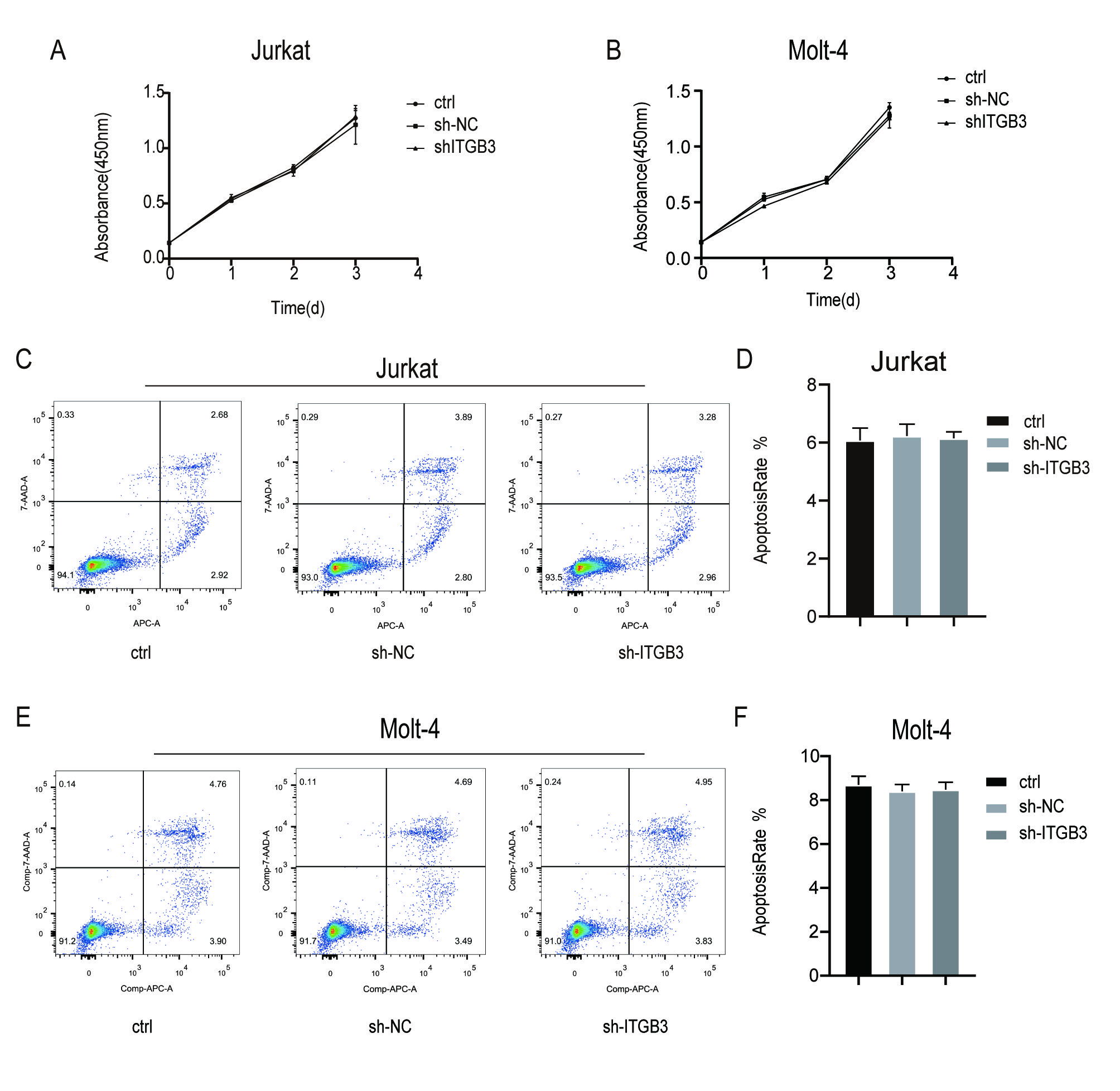

Supplement: Supplemental Material [file KCAM_A_2191913_SM9144.zip › Supplementary Figure4_for review.jpg]

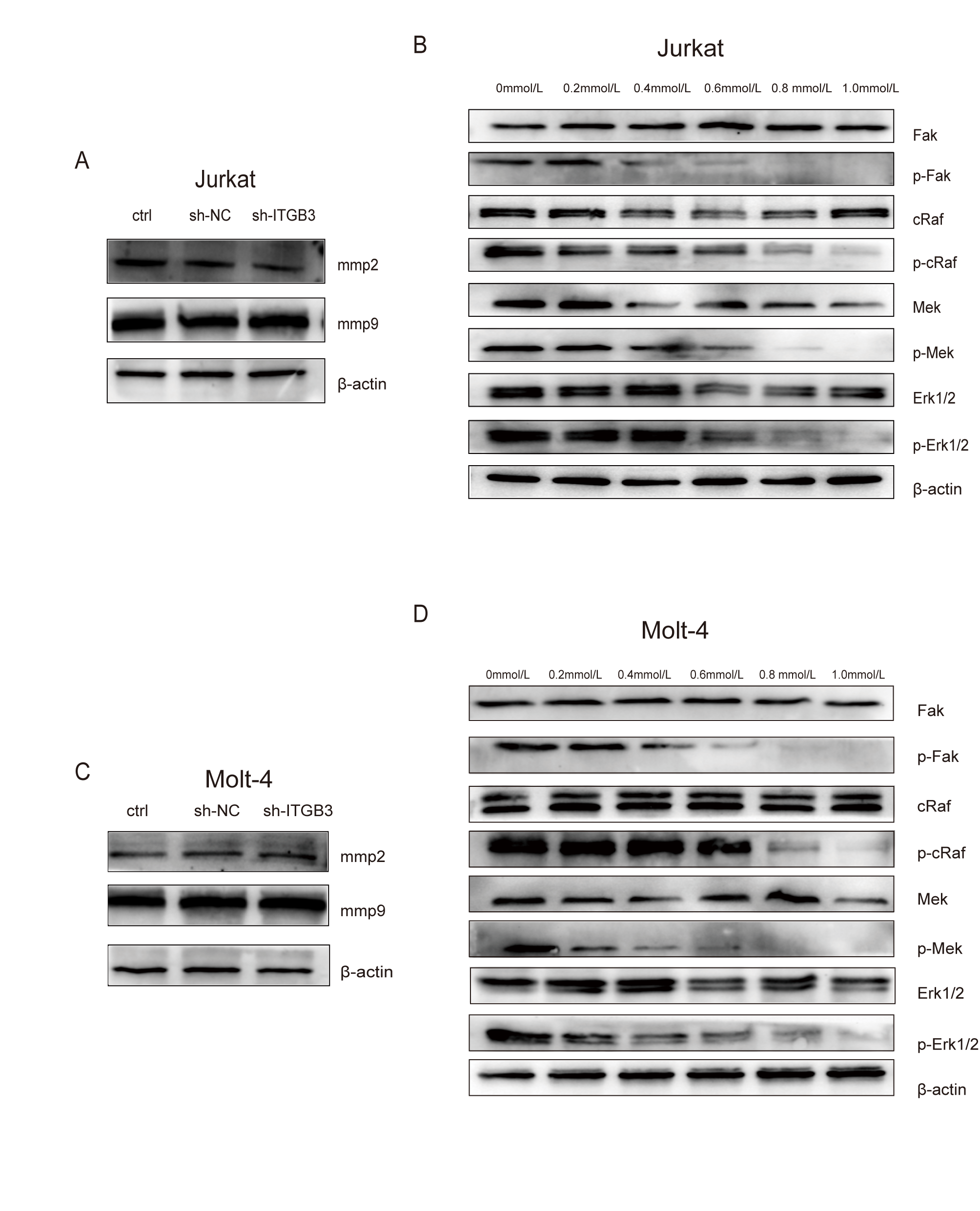

Supplement: Supplemental Material [file KCAM_A_2191913_SM9144.zip › Supplementary Figure5_for review_1.tif]

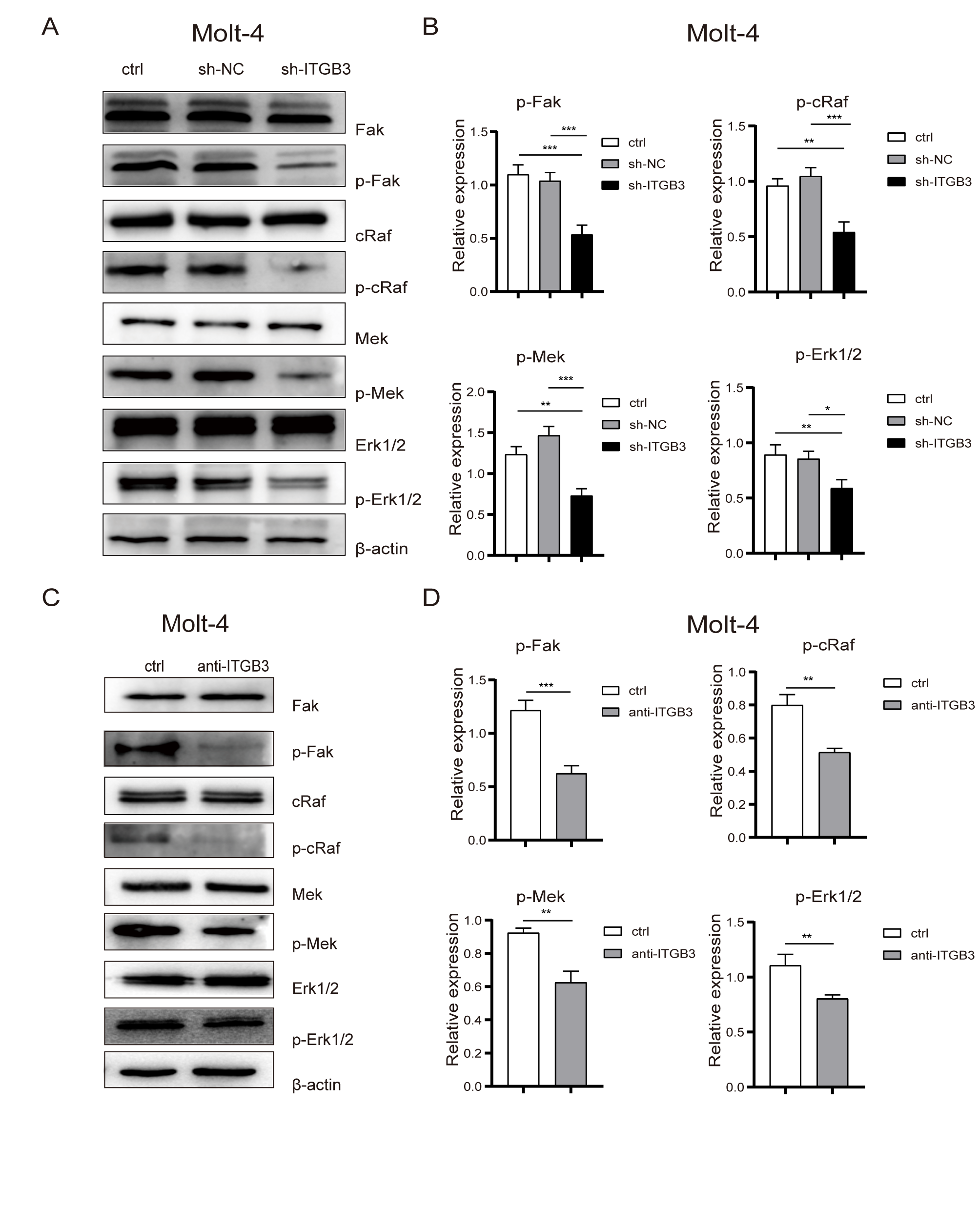

Supplement: Supplemental Material [file KCAM_A_2191913_SM9144.zip › Supplementary Figure6_for review_1.tif]
